# Enhancement of Jasmonate-Mediated Antiherbivore Defense Responses in Tomato by Acetic Acid, a Potent Inducer for Plant Protection

**DOI:** 10.3389/fpls.2019.00764

**Published:** 2019-06-07

**Authors:** Daoqian Chen, Min Shao, Shaozhi Sun, Tingting Liu, Hao Zhang, Ningning Qin, Rensen Zeng, Yuanyuan Song

**Affiliations:** ^1^Key Laboratory of Ministry of Education for Genetics, Breeding and Multiple Utilization of Crops, College of Crop Science, Fujian Agriculture and Forestry University, Fuzhou, China; ^2^Institute of Crop Resistance and Chemical Ecology, College of Life Sciences, Fujian Agriculture and Forestry University, Fuzhou, China

**Keywords:** defense priming, induced defense, acetic acid, jasmonate pathway, tomato, *Spodoptera litura*

## Abstract

Acetic acid (AA) has been proved as a chemical that could prime the jasmonic acid (JA) signaling pathway for plant drought tolerance. In this study, the capability of AA for priming of tomato defense against a chewing caterpillar *Spodoptera litura* and its underlying molecular mechanism were evaluated. AA pretreatment significantly increased tomato resistance against *S. litura* larvae. Upon larval attack, tomato plants pretreated with AA exhibited increased transcript levels of defense-related genes and elevated activities of polyphenol oxidase (PPO) and peroxidase (POD), and accumulation of protease inhibitor. Moreover, AA pretreatment resulted in upregulated transcription of JA biosynthesis genes and elevated JA accumulation in tomato seedlings upon insect attack. Furthermore, an apparent loss of AA-induced resistance was observed in a JA pathway-impaired mutant *suppressor of prosystemin-mediated responses8* (*spr8*). These results indicate that AA enhances jasmonate-mediated antiherbivore defense responses in tomato. This raises the possibility of use of AA, a basic and simple biochemical compound, as a promising inducer for management of agricultural pests.

## Introduction

Insect pests are one of the most important factors limiting the productivity of agricultural crops worldwide, which cause an estimate of 10–20% reduction in crop yields by both direct damage and indirect transmission of plant diseases (Ferry et al., 2006; Douglas, 2018). To feed the increasing human population, it is an agent need to reduce insect pest damage. The demand for novel sustainable strategies to control insect pests is particularly urgent since wide use of chemical insecticides, which have been the mainstay of crop protection against insects over the last 50 years, has resulted in increasing insect resistance, environmental toxicity and concerns for human health (Douglas, 2018).

In nature sessile plants have evolved various strategies to defend themselves against insect herbivores. Upon herbivore attack, plants initiate defense responses by activation of jasmonate, ethylene and salicylic acid (SA) signaling pathways, induction of defense-related genes, and production of defense compounds (Mithofer and Boland, 2012; Douglas, 2018). Defense priming is a unique physiological process by which a plant prepares to more quickly or aggressively respond to future biotic or abiotic stress (Frost et al., 2008; Mauch-Mani et al., 2017) after previous exposure to a stimulus. The primed plants are able to induce more effective defense responses upon subsequent attack with minimal associated metabolic costs (Conrath et al., 2015). Anti-herbivore defense priming could be initiated by environmental cues, such as prior insect damage, insect oviposition, pathogen challenge, and volatile emissions from neighboring plants, that reliably indicates an increased probability of a forthcoming attack (Peng et al., 2011; Kim et al., 2012; Rasmann et al., 2012; Mauch-Mani et al., 2017). The primed state in plants can also be provoked by various natural and synthetic compounds, such as jasmonic acid (JA), SA, β-aminobutyric acid (BABA), and silicate (Si) (Ye et al., 2013; Conrath et al., 2015). Seed treatments with JA and BABA lead to increased resistance against herbivory by spider mites, caterpillars, and aphids, and against fungal pathogens in tomato (Worrall et al., 2012). Thiamine is capable of inducing rapid and effective defense responses to impede various invading microbial pathogens (Ahn et al., 2007; Asensi-Fabado and Munne-Bosch, 2010) and rice root-knot nematode (*Meloidogyne graminicola*) infection (Huang et al., 2016). Exogenous Si application primed rice defense against caterpillar rice leaffolder (*Cnaphalocrocis medinalis*) and root-knot nematode (Ye et al., 2013; Zhan et al., 2018).

Organic acid metabolism is of fundamental importance in plants for several major biochemical processes, including energy production, photosynthesis, amino-acid biosynthesis, nutrient uptake, detoxification of heavy metals, and soil ecology (Lopez-Bucio et al., 2000; Hawrylak-Nowak et al., 2015). It has been shown that citrate and fumarate, two major organic acids of the tricarboxylic acid cycle, are able to prime *Arabidopsis* defense against the bacterial pathogen *Pseudomonas syringae* pv. tomato DC3000 (Balmer et al., 2018). Acetic acid (AA) is a ubiquitous low molecular weight organic acid. Akin to other organic acid compounds, exogenously applied AA reduces cadmium phytotoxicity in sunflower plants (Hawrylak-Nowak et al., 2015). AA has also been used for weed control in potato production at 20% concentration (Ivany, 2010). In human cells, exogenous AA treatment increases intracellular pH and elevates histone acetylation associated with cell proliferation (McBrian et al., 2013). Recently, Kim et al. (2017) reported that exogenous AA promoted *de novo* JA synthesis and enrichment of histone H4 acetylation, which initiated the priming of the JA signaling dependent plant drought tolerance. Given the essential role of JA signaling pathway in plant responses to chewing insects (Howe et al., 2018), these results strongly suggest that AA could be a potential chemical inducer for antiherbivore defense priming.

The aim of this study was to determine the possibility of antiherbivore defense priming by AA and its underlying molecular mechanisms in tomato resistance against *Spodoptera litura*, a notorious pest worldwide that causes enormous losses to many economically important crops (Cheng et al., 2017). Our results demonstrate that AA enhanced plant resistance against the chewing insect herbivore *S. litura*. AA pretreatment of tomato seedlings resulted in elevated JA-mediated transcriptional responses, promoted defense-related enzymatic activities and increased levels of JA accumulation following *S. litura* attack. Furthermore, an apparent loss of AA-induced *S. litura* resistance was observed in the JA pathway-impaired mutant *suppressor of prosystemin-mediated responses8* (*spr8*). The present study suggests that AA, a basic and simple biochemical compound, may serve a novel priming agent for insect pest control in crop production.

## Materials and Methods

### Plant Growth and AA Treatment

Tomato (*Solanum lycopersicum* L.) cv. Fenfan No. 1, a JA biosynthesis defective mutant *spr8* and the corresponding wild-type (cv. Castlemart, CM) (Yan et al., 2013) were used in this study. Tomato plants CM and *spr8* were kindly provided by Prof. Chuanyou Li of the Institute of Genetics and Developmental Biology, the Chinese Academy of Sciences. Tomato seeds were sterilized with 1% sodium hypochlorite for 10 min and then washed with distilled water four times before sowing into autoclaved gardening soil (Pindstrup Blond Gold, Pindstrup Mosebrug A/S, Denmark). After germination, the uniform healthy seedlings were transplanted into a plastic container (Diameter × Height: 110 × 140 mm) filled with gardening soil. Tomato seedlings were grown in growth chambers and maintained under 16 h of light at 28°C and 8 h of dark at 18°C and 60% relative humidity.

In a preliminary experiment, different concentrations (0, 1, 5, 10, 20, and 50 mM) of AA solution (100 ml, the amount of normal watering) were applied to tomato seedlings and the larval mass gain was assessed. Pretreatments with 10, 20 and 50 mM AA significantly enhanced tomato resistance against *S. litura*, but the growth of seedlings were inhibited by pretreatments with >20 mM AA. Therefore, the concentration of 10 mM was chosen for further studies. Three weeks after transplanting, 100 ml solution of 10 mM AA (++AA) or distilled water (−AA, control) were supplied to the soil and the plants were grown for 6 days. After removing the treatment solutions by using a paper towel under the bottom of the pot, 20 plants for each treatment were subjected to larval inoculation.

### Herbivore Treatment

Tobacco cutworm *S. litura* (SL) was used to infest tomato plants. The SL larvae were reared on an artificial diet according to Wang et al. (2015) and maintained in an insectary at 23–26°C, 16 h/8 h (day/night) and 65–70% relative humidity. Five weighted homogenously second instar larvae (∼5 mg each larva) were placed on each of two leaves (leaf 4 and 5, the youngest fully expanded leaves), and the leaves were caged with gauze bags (80 mesh, Length × width: 200 × 150 mm) (+SL), and corresponding leaves of control plants were caged in the same way (-SL). After 2 days, the larvae were removed and weighed by electronic balance (0.1 mg, ATX224, Shimadzu, Kyoto, Japan). Each treatment included 20 plants. The experiments were biologically repeated three times with similar trends of SL weight gain (20 replicates). In the last experiment, the caged local leaves were harvested, the SL-attacked leaf tissues of infested plants and corresponding leaf tissues of control plants were sampled. The sampled leaf tissues from five plants were pooled together for a single replicate and stored at −80°C for analyses of enzyme activity, JA content and gene expression with four replicates.

### Enzyme Activity Assays and PI Analysis

Peroxidase (POD) activity was measured by a colorimetric assay following the change of absorption at 420 nm due to guaiacol oxidation (Han et al., 2016). Polyphenol oxidase (PPO) activity was assayed with catechol as substrate following the method of Cai et al. (2008). Accumulation of proteinase inhibitor II (PI-II) was measured using the classical radial immunodiffusion assay (Yan et al., 2013). Four replicates were conducted for each measurement.

### JA Analysis

Frozen leaves (200 mg) were subjected to quantification of JA by UPLC-MS/MS using single SPE purification and isotope dilution as described by Fu et al. (2012). Each treatment included four replicates.

### Gene Expression Analysis

For qRT-PCR analysis, leaf tissues were harvested and frozen in liquid nitrogen for RNA extraction. RNA extraction and qRT-PCR analysis were performed as previously described (Song et al., 2013). Four replicates each treatment were used for qRT-PCR analyses. Expression levels of target genes were normalized to those of the tomato *Actin2* gene. Primers used to quantify gene expression levels are listed in Supplementary Table S1.

### Statistical Analysis

Statistical analysis was performed using SPSS statistical software (Version 190 for Windows, SPSS, Chicago, IL, United States). The data of different treatments were firstly checked for normality (Shapiro–Wilk test) and variance homogeneity (Levene test). ANOVA was used to assess the main effects of AA treatment and experiment time on the larval weight gain. The main effects and interactions of AA treatment, herbivore treatment and/or genotype on the rest of parameters were evaluated by two-way or three-way ANOVA. Differences among means were compared using a Tukey *post hoc* test at *P* ≤ 0.05. *F* and *P*-values of ANOVA analysis of variance were listed in Supplementary Table S2.

## Results

### AA Enhances Tomato Resistance Against *S. litura*

**FIGURE 1:**
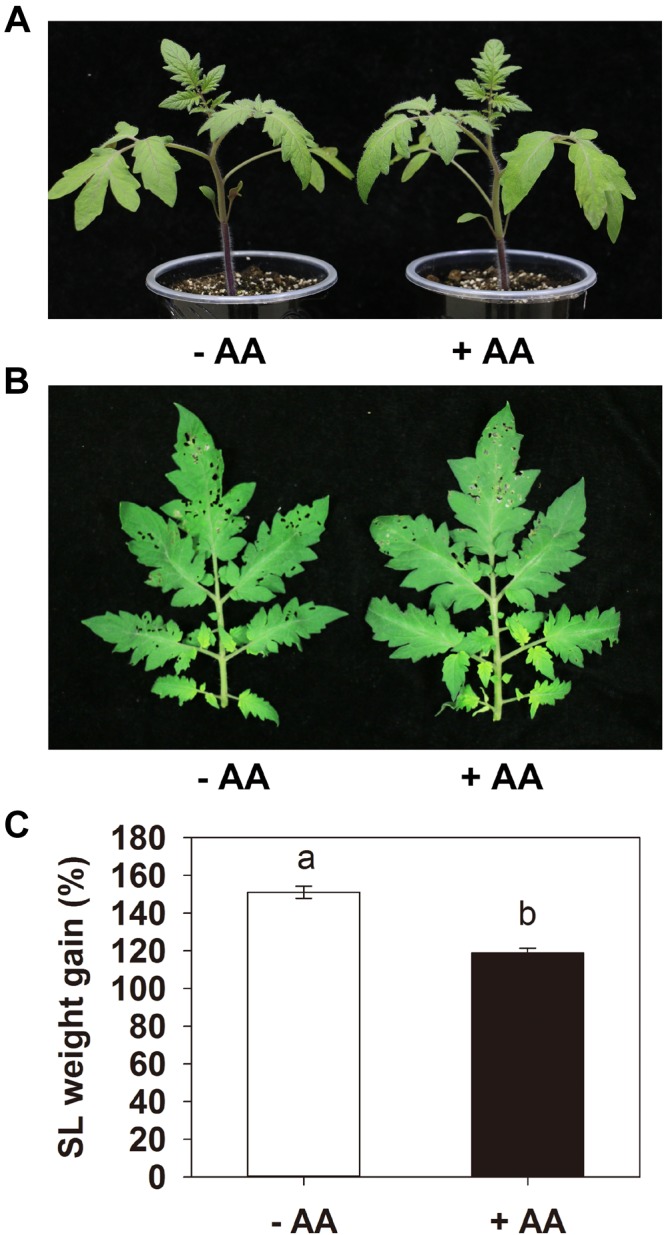
Acetic acid enhances tomato resistance against *Spodoptera litura* (SL). Tomato plants were treated with water (–AA) or 10 mM acetic acid (+AA) for 6 days and then inoculated with 10 s instar larvae (5 per leaf). **(A)** Tomato plants with or without AA application. **(B)** Representative leaves of plants pretreated with or without AA after 2 days of larval feeding. **(C)** Weight gain of larvae at the end of SL feeding trial. Data show the mean ± SE (*n* = 20). The experiments were repeated three times with similar trends and all data were combined. Different letters above bars indicate statistically significant differences between treatments (Tukey’s multiple range test, *P* ≤ 0.05).

Pretreatment with 10 mM AA did not obviously affect the tomato seedling growth (Figure 1A). After 2 days of larval feeding, the leaves of plants treated with water were severely damaged, while the plants treated with AA showed significantly lower leaf damage (Figure 1B). The weight gain of the larvae fed on plants AA-pretreated was significantly lower than that on plants treated with water in all three independent experiments. A representative one is shown in Figure 1C, the larvae fed on plants treated with water showed 149.4% weight gain 2 d after insect inoculation, whereas larvae fed on AA-pretreated plants showed only 98.6% weight gain (*P* = 0.003). The results indicate that AA can enhance tomato resistance against *S. litura*.

### AA Elevates the Activities of Defense-Related Enzymes and Accumulation of Protease Inhibitors Upon Insect Attack

**FIGURE 2:**
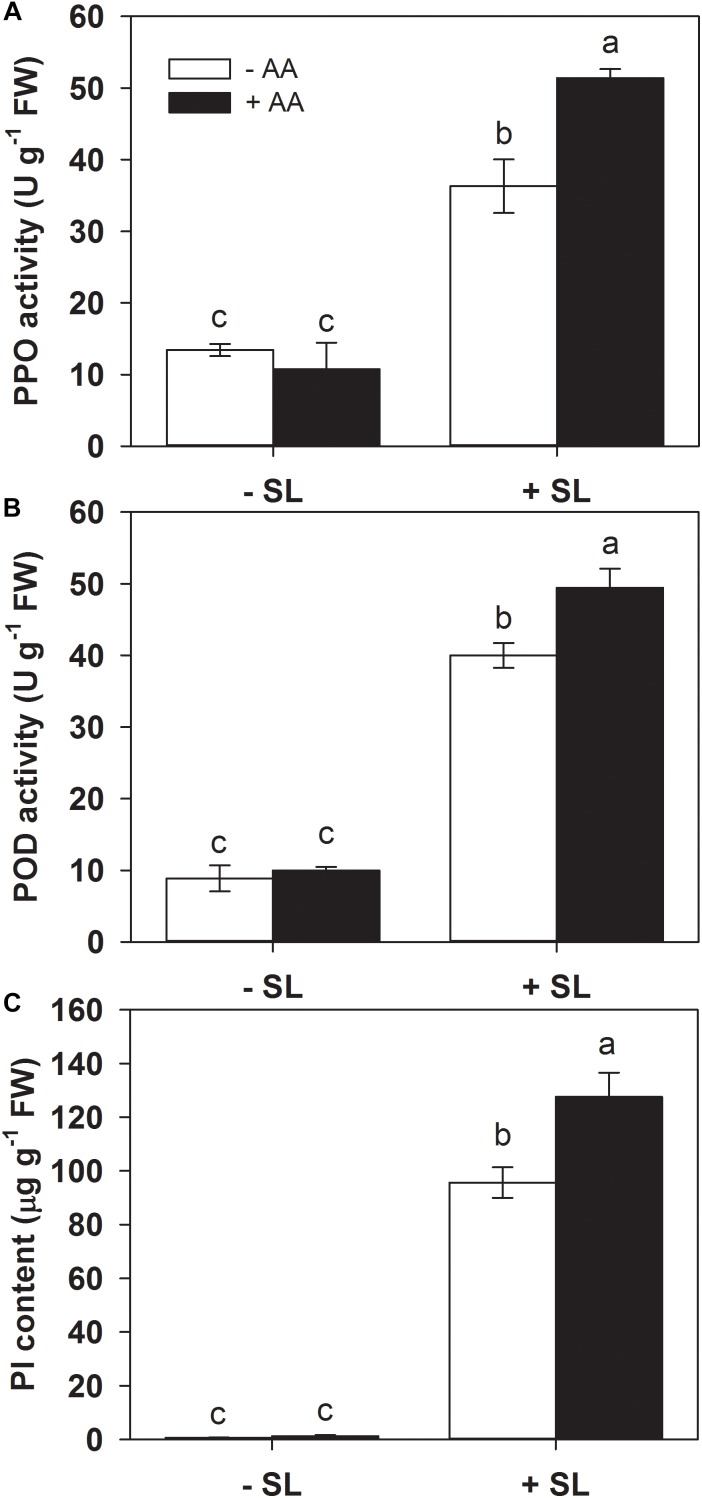
Activity levels of PPO **(A)**, POD **(B)**, and PI **(C)** in attacked leaves by *S. litura* (SL) of tomato plants pretreated with or without 10 mM AA after 2 days of SL infestation. Data show the mean ± SE (*n* = 4). Different letters above bars indicate statistically significant differences between treatments (Tukey’s multiple range test, *P* ≤ 0.05).

Defense-related enzymes such as PPO and POD, as well as protease inhibitors (PI), represent important components of plant inducible defense responses (Ye et al., 2013). To further examine the potential effects of AA pretreatment on plant defense responses, the activities of PPO and POD, as well as PI accumulation in the remaining leaf tissues after insect infestation were evaluated (Figure 2). AA pretreatment by itself did not significantly alter the activities of PPO and POD, or PI levels. In plants without AA pretreatment, SL attack induced PPO, POD activities, and PI accumulation by 1.7-, 3.5-, and 135-fold, respectively, compared with those without insect infestation. However, the magnitude of these inductions by SL attack in AA-pretreated plants were higher than that in untreated plants (Figure 2 and Supplementary Table S2). These results indicate that AA application promoted inducibility of defense-related enzymes and PI.

### AA Improves the Local Transcriptional Responses of Defense-Related Genes Upon Insect Attack

**FIGURE 3:**
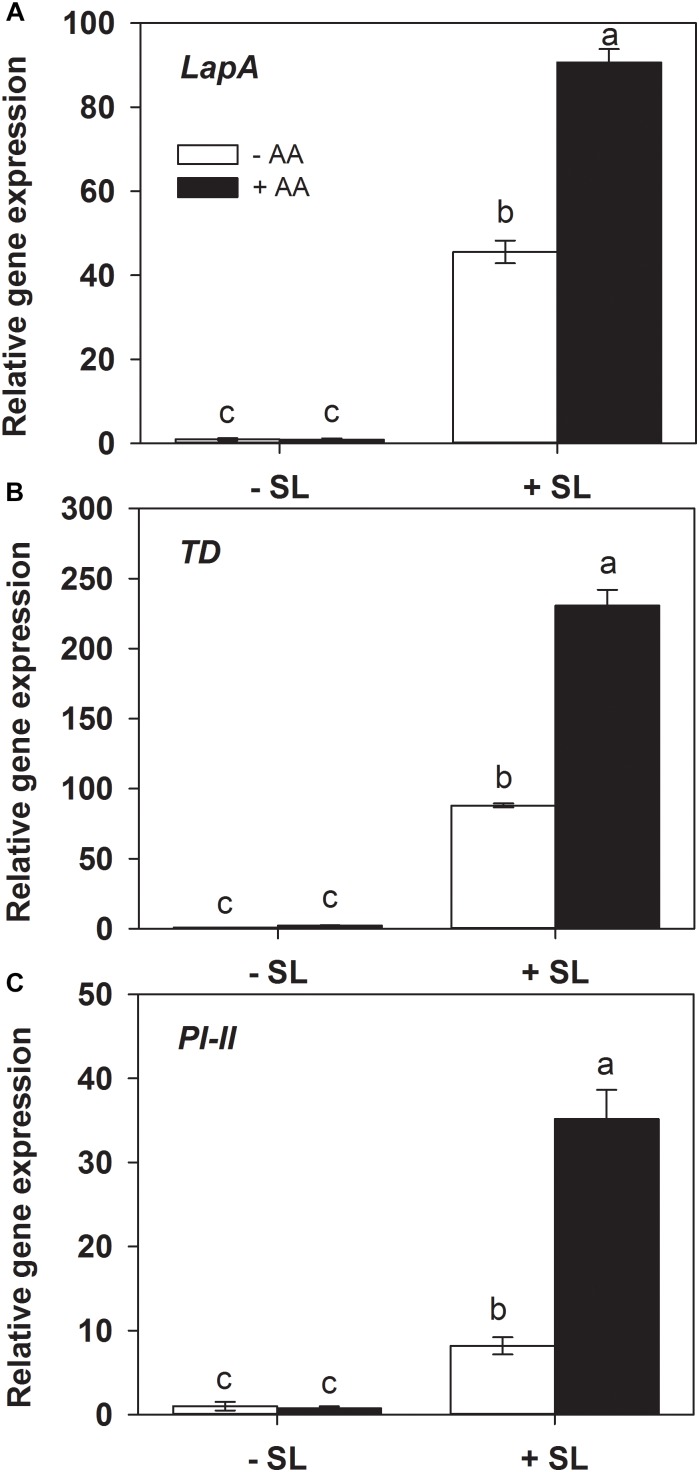
Transcript levels of defense-related genes *leucine amino peptidase A* (*LapA*, **A**), *threonine deaminase* (*TD*, **B**), and *proteinase inhibitor* (*PI-II*, **C**) in attacked leaves by *S. litura* (SL) of tomato plants pretreated with or without 10 mM AA after 2 days of SL infestation. Data show the mean ± SE (*n* = 4). Different letters above bars indicate statistically significant differences between treatments (Tukey’s multiple range test, *P* ≤ 0.05).

Protease inhibitors (Green and Ryan, 1972), threonine deaminase (TD, Chen et al., 2005) and leucine amino peptidase A (LapA, Fowler et al., 2009) play a key role in plant defense against insect herbivory. The transcripts levels of the related genes were also examined by quantitative RT-PCR. In absence of insect herbivory, AA pretreatment alone did not induce transcript levels of all three genes (Figure 3). In plants without AA pretreatment, insect infestation induced the transcript levels of *PI-II*, *TD*, and *LapA* by 10-, 90-, and 50-fold, respectively, relative to those without insect infestation. However, the magnitude of inductions of the three genes by SL attack in AA-pretreated plants were significantly higher than that in untreated plants (Supplementary Table S2). These results indicate that AA application enhanced the transcriptional responses of defense-related genes upon insect attack.

### AA Promotes the Local Transcriptional Responses of JA Biosynthesis Genes and JA Accumulation Upon Insect Attack

**FIGURE 4:**
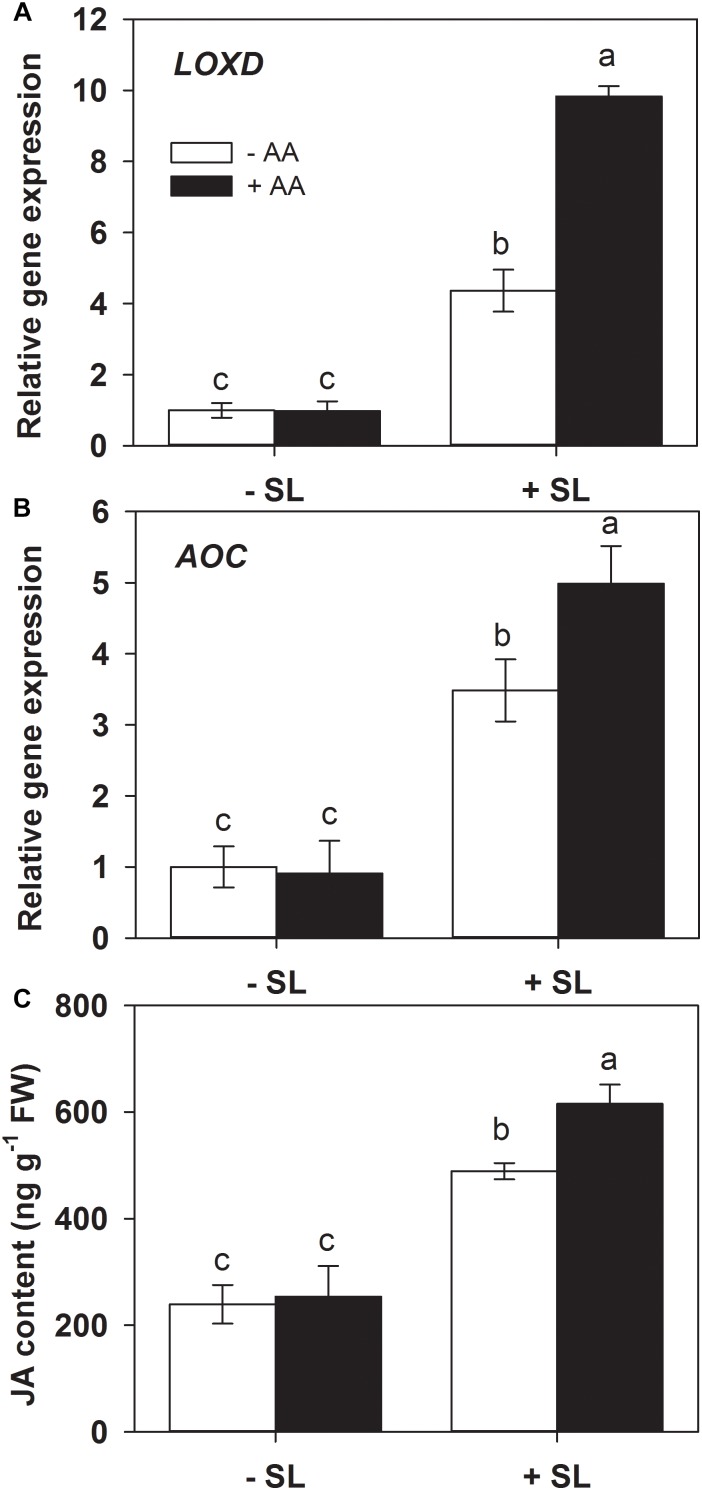
Transcript levels of *lipoxygenase D* (*LOXD*, **A**) and *allene oxide cyclase* (*AOC*, **B**), and JA content **(C)** in attacked leaves by *S. litura* (SL) of tomato plants pretreated with or without 10 mM AA after 2 days of SL infestation. Data show the mean ± SE (*n* = 4). Different letters above bars indicate statistically significant differences between treatments (Tukey’s multiple range test, *P* ≤ 0.05).

Jasmonic acid is well known to play a central role in mediating plant defense responses against insect herbivores (Browse, 2009; Howe et al., 2018). Lipoxygenase D (LOXD) and allene oxide cyclase (AOC) are two key enzymes in JA biosynthesis (Han, 2017). Given the potential priming effects of AA on the JA signaling pathway (Kim et al., 2017), the influence of AA on SL infestation-induced JA biosynthesis was examined by monitoring the accumulation of endogenous transcript levels of *LOXD* and *AOC*, and JA content in AA-treated and untreated plants exposed to SL infestation (Figure 4). AA treatment alone had no significant effect on endogenous *LOXD* and *AOC* transcript levels. In plants without AA pretreatment, mRNA levels of *LOXD* and *AOC* were increased 3.2- and 2.6-fold by insect infestation, respectively. However, the magnitude of transcriptional responses of both genes in AA-treated plants was significantly higher relative to untreated plants (Figure 4 and Supplementary Table S2).

Similarly, JA accumulation did not display significant differences before insect infestation between AA-treated and untreated control plants. However, after SL infestation, JA level in AA-treated plants was significantly higher than that in untreated plants (Figure 4C, *P* = 0.005). These results suggest that AA pretreatment can enhance the JA biosynthesis and accumulation upon insect attack.

### AA-Mediated Resistance Is Dependent on the JA Signaling Pathway

**FIGURE 5:**
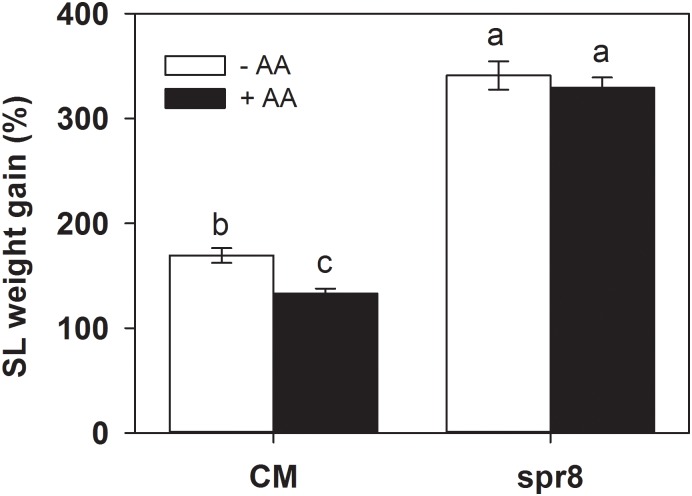
Weight gain of *S. litura* (SL) larvae fed on wild-type (CM, cv. Castlemart) and *spr8* mutant plants of tomato pretreated with 10 mM AA after 2 days of *S. litura* (SL) infestation. Data show the mean ± SE (*n* = 40). Different letters above bars indicate statistically significant differences between treatments (Tukey’s multiple range test, *P* ≤ 0.05). The experiments were repeated three times and a representative replicate is shown.

**FIGURE 6:**
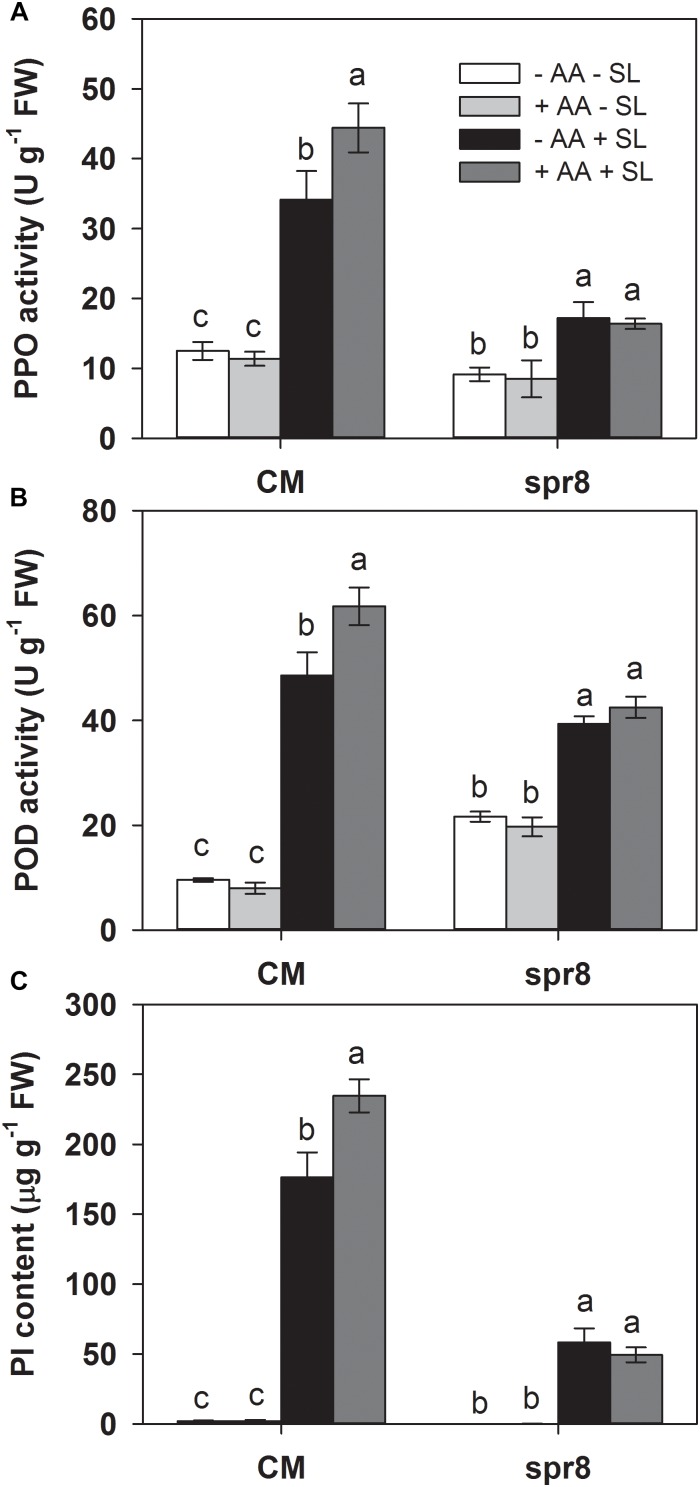
Activity levels of PPO **(A)**, POD **(B)**, and PI **(C)** in attacked leaves by *S. litura* (SL) of wild-type (CM, cv. Castlemart) and *spr8* mutant plants of tomato pretreated with or without 10 mM AA after 2 days of SL infestation. Data show the mean ± SE (*n* = 4). Different letters above bars indicate statistically significant differences between treatments (Tukey’s multiple range test, *P* ≤ 0.05).

To further investigate the role of the JA pathway in AA-induced insect resistance, the JA biosynthesis mutant (*spr8*), which is defective in the catalytic domain of LOXD, a chloroplast-localized lipoxygenase involved in JA biosynthesis (Yan et al., 2013), and the corresponding wild-type (CM) were used to study their differential defense responses to insect herbivory and AA pretreatment. The *spr8* mutant plants showed significantly higher sensitivity to *S. litura* infestation. After 2 days of insect infestation, the leaves of *spr8* plants were more severely damaged and *S. litura* larvae fed on mutant plants gained significantly more weight than those fed on CM plants (Figure 5). Moreover, in contrast to the substantial increase of PPO, POD activities, and PI protein accumulation induced by *S. litura* infestation in CM plants, PPO, POD activities, and PI protein levels remained unchanged or increased only marginally in *S. litura-*infested *spr8* mutant plants (Figure 6 and Supplementary Table S2). These results demonstrate that JA plays an important role in tomato resistance against *S. litura* infestation (Browse, 2009; Yan et al., 2013).

Importantly, *spr8* mutant plants did not respond to AA pretreatment when exposed to *S. litura* infestation (Figure 5, 6). All larvae fed on mutant plants pretreated with or without AA treatment gained huge but similar increase in larval weight (Figure 5). Furthermore, the AA-mediated enhancement of PPO, POD activities, and PI induction after insect attack was apparently lost in the *spr8* mutant plants. These results indicate that the AA-mediated enhancement of tomato resistance against insect herbivore attack occurred in a JA-dependent manner.

## Discussion

Plants use both constitutive and inducible defensive strategies against insect herbivores (Mithofer and Boland, 2012). Inducible defenses allow plants to manage energy reserves more efficiently by activation of defense only when needed (Mithofer and Boland, 2012; Douglas, 2018). Defense priming in plants refers to quicker and stronger defense responses to enemy attack after initial exposure of plants to one stimulus (Mauch-Mani et al., 2017; Schillheim et al., 2018). The stimuli can be either beneficial microbes, microbial pathogens, insect herbivores, or certain chemicals, which induce plants to enter a special “ready to fight” physiological state called “primed state” (Bernsdorff et al., 2016; Coppola et al., 2017a; Mauch-Mani et al., 2017; Schillheim et al., 2018). The primed plants show enhanced resistant levels when attacked (Frost et al., 2008; Coppola et al., 2017b). Certain chemicals have been shown to be able to prime plant defenses (Bernsdorff et al., 2016; Mauch-Mani et al., 2017). Currently most studies on antiherbivore defense priming focus on priming by herbivore-induced volatile compounds (Engelberth et al., 2004; Kim and Felton, 2013; Erb et al., 2015). The present study suggested that AA, a basic and simple biochemical compound, might prime defense responses in tomato against insect herbivores. This raises the possibility of use of AA as a promising alternative for management of agricultural pests.

Balmer et al. (2018) showed that citrate and fumarate, two major organic acids of the tricarboxylic acid cycle, can prime *Arabidopsis* against the bacterial pathogen *P. syringae* pv. tomato DC3000. AA has also been proven as a chemical that could prime plant drought tolerance (Kim et al., 2017). In the present study, AA pretreatment itself did not influence the tomato seedling growth and affect transcription of the tested defense-related genes, and activities of defense-related enzymes. However, insect attack provoked much stronger induction of these defense responses in AA-treated plants (Figure 2, 3). Our results provide strong evidence of the role of AA in priming defense responses to herbivore attack in tomato, and consequently, AA pretreated plants showed higher resistance against herbivore infestation. More research efforts are needed to verify if AA application influences tomato fruit production, ripening as well as fruit taste. Further cellular and molecular studies are also required to confirm the defense priming effects of AA against herbivory.

The JA signaling pathway plays a crucial role in mediating antiherbivore defense responses in plants (Howe and Jander, 2008; Pieterse et al., 2012; Howe et al., 2018). It is generally believed that wounding and insect attack lead to the activation of defense gene expression by increasing endogenous levels of JA and related pentacyclic oxylipins that are derived from the linolenic acid (Farmer and Ryan, 1992; Schaller, 2001; Han, 2017; Howe et al., 2018). The JA pathway has been linked to antiherbivore defense priming by various priming signals (Conrath et al., 2015). The JA signaling is involved in defense priming by volatile organic compounds (VOC) (Engelberth et al., 2004; Kessler et al., 2006; Paschold et al., 2006) and plant beneficial microbes (Verhagen et al., 2004; Song et al., 2013). Recent study by Kim et al. (2017) showed that AA could prime the JA signaling pathway for plant drought tolerance. In the present study, AA treatment alone did not affect either transcripts of key enzyme genes of the JA biosynthesis pathway or JA content. However, in presence of *S. litura* infestation, induction of both JA biosynthesis genes and JA level in AA-treated plants was significantly higher than that in untreated plants (Figure 4). These results suggest that the JA pathway is involved in the AA-mediated anti-herbivore resistance.

Lipoxygenase catalyzes the key initial reaction in the JA biosynthesis pathway, which inserts molecular oxygen into position 13 of α-linolenic acid (Christensen et al., 2013; Han, 2017). The tomato mutant spr8 that is defective in *TomLoxD* exhibits a series of JA-dependent immune deficiencies, including the inability to express wound responsive genes, abnormal development of glandular trichomes, and severely compromised resistance to insect herbivory attack and necrotrophic pathogen infection (Yan et al., 2013). Use of *spr8* mutant plants made it possible to identify the essential role the JA signaling pathway in AA-enhanced defense in tomato plants. In this study, the *spr8* mutant plants were extremely susceptible to insect infestation and AA pretreatment did not affect their anti-herbivore resistance (Figure 5). Moreover, in contrast to the enhancement of the activities of defense-related enzymes by AA in CM, AA pretreatment had no obvious influence on induction of these defense responses upon insect attack in the *spr8* mutant plants (Figure 6). The results obtained here indicated that the JA pathway is required for AA-enhanced defense against insect herbivory.

Although defense priming has been considered a key process in various types of systemic plant immunity, the underlying molecular mechanisms remain elusive (Conrath et al., 2015; Martinez-Medina et al., 2016). One hypothesis proposed that epigenetic modifications, including DNA methylation, histone modification or chromatin remodeling, would prime defense genes for faster and stronger transcription (Martinez-Medina et al., 2016; Mauch-Mani et al., 2017). It is reported that chemical benzothiadiazole (BTH) primed the expression of Arabidopsis *WRKY* transcription factors by inducing histone modifications of H3K4me3, H3K4me2, and acetylation of H3K9 (H3K9ac), H4K5ac, H4K8ac, and H4K12ac in their promoters (Jaskiewicz et al., 2011). Moreover, MeJA primed plants for increased expression of defense-related gene *OsBBPI* encoding a Bowman–Birk protease inhibitor responsive to wounding and JA, by modulating histone modifications of H3K4me3 and H3K9ac in the promoter region of *OsBBPI* (Bertini et al., 2018). Kim et al. (2017) suggested that the exogenous AA is converted to acetyl-CoA and is used as a substrate for histone acetylation in *Arabidopsis*. Furthermore, ChIP-seq analysis showed that histone H4 acetylation (H4ac) of the gene body region was enriched genome-wide by addition of 10 mM AA. Importantly, the JA biosynthesis genes, such as *LOX6*, *AOC4* and *allene oxide synthase* (*AOS*), and *MYC2*, coding the key transcriptional activator in the JA signaling pathway (Lorenzo et al., 2004; Han, 2017), are involved in the H4ac-enriched genes (Kim et al., 2017). Further cellular and molecular studies are needed to identify the underlying molecular mechanisms of AA-induced defense against herbivory.

In conclusion, this study demonstrates that AA can enhance tomato resistance against *S. litura*, and this AA-enhanced defense may be associated with priming of the host plants for an efficient activation of defense responses upon herbivore attack. The study also indicates that the JA pathway is involved in the AA-mediated defense. Despite further studies are needed to confirm the defense priming effects of AA against herbivory and clarify the underlying molecular mechanisms, this study indicate that AA may serve as a novel eco-friendly inducer for pest management in crop production.

## Author Contributions

DC, RZ, and YS planned the experiments and wrote the manuscript. DC and MS conducted the experiments, collected and analyzed the data. HZ, SS, NQ, and TL helped in data collection and drafted the preparation.

## Conflict of Interest Statement

The authors declare that the research was conducted in the absence of any commercial or financial relationships that could be construed as a potential conflict of interest.
