# Supplementary material for: Enhancement of Jasmonate-Mediated Antiherbivore Defense Responses in Tomato by Acetic Acid, a Potent Inducer for Plant Protection
Source: Front Plant Sci. 2019 Jun 7;10:764. doi: 10.3389/fpls.2019.00764 (PMC6566139; doi:10.3389/fpls.2019.00764)
Supplement: Supplementary file 1 [file Data_Sheet_1.doc]

***Supplementary Material*：**

**Enhancement of jasmonate-mediated antiherbivore defense responses in tomato by acetic acid, a potent inducer for plant protection**

**Daoqian Chen^#^, Min Shao^#^, Shaozhi Sun, Tingting Liu, Hao Zhang, Ningning Qin, Yuanyuan Song^*^, Rensen Zeng**

* **Correspondence:**

Dr. Yuanyuan Song, Email: yyuansong@163.com

Institute of Crop Resistance and Chemical Ecology

College of Crop Science

Fujian Agriculture and Forestry University

Fuzhou 350002, P.R. China

**Running title:** Acetic acid enhanced tomato antiherbivore defense responses

**Supplementary TABLE 1 | Genes and oligonucleotides used in the real-time quantitative PCR experiment.**

| **Gene** | **Primer** |
| --- | --- |
| *Actin2* | F: 5'-TTGCTGACCGTATGAGCAAG-3' |
|  | R: 5'- GGACAATGGATGGACCAGAC-3' |
| *LapA* | F: 5'-ATCTCAGGTTTCCTGGTGGAAGGA-3' |
|  | R: 5'-AGTTGCTATGGCAGAGGCAGAG-3' |
| *TD* | F: 5'-AGCTCAAACACACGCGCTGGA-3' |
|  | R: 5'-AACCCCCACCACCAACAGGT-3' |
| *PI-II* | F: 5'-CTTCTTCCAACTTCCTTTG-3' |
|  | R: 5'-TGTTTTCCTTCGCACATC-3' |
| *LOXD* | F: 5'-ACTCATCAGCACCGACATCG-3' |
|  | R: 5'-ACTCTCCAGAAAGAACTCCTGC-3' |
| *AOC* | F: 5'-CTCGGAGATCTTGTCCCCTTT-3' |
|  | R: 5'-CTCCTTTCTTCTCTTCTTCGTGCT-3' |

**Supplementary TABLE 2 | Summary (F and P values) of analysis of variance (ANOVA).** After tests of normality and varience homogeneity, ANOVA was used to assess the main effects of AA treatment and experiment time on the larval weight gain. The main effects and interactions of AA treatment, herbivore treatment and/or genotype on the rest of parameters were evaluated by two-way or three-way ANOVA. df: degrees of freedom.

|  | **Effect** | **df** | **F value** | **P value** |
| --- | --- | --- | --- | --- |
| SL weight gain (%) | AA Treatment (AA) | 1 | 9.456 | 0.003 |
|  | Experiment time (T) | 2 | 13.298 | 0.000 |
| PPO activity | AA Treatment (AA) | 1 | 15.397 | 0.002 |
|  | Herbivore Treatment (SL) | 1 | 404.252 | 0.000 |
|  | AA * SL | 1 | 31.583 | 0.000 |
| POD activity | AA Treatment (AA) | 1 | 16.694 | 0.002 |
|  | Herbivore Treatment (SL) | 1 | 745.850 | 0.000 |
|  | AA * SL | 1 | 10.405 | 0.007 |
| PI activity | AA Treatment (AA) | 1 | 9.559 | 0.009 |
|  | Herbivore Treatment (SL) | 1 | 446.559 | 0.000 |
|  | AA * SL | 1 | 9.016 | 0.011 |
| Transcript levels of *LaPA* | AA Treatment (AA) | 1 | 116.571 | 0.000 |
|  | Herbivore Treatment (SL) | 1 | 1036.012 | 0.000 |
|  | AA * SL | 1 | 117.248 | 0.000 |
| Transcript levels of *TD* | AA Treatment (AA) | 1 | 47.874 | 0.000 |
|  | Herbivore Treatment (SL) | 1 | 229.249 | 0.000 |
|  | AA * SL | 1 | 46.112 | 0.000 |
| Transcript levels of *PI-II* | AA Treatment (AA) | 1 | 345.355 | 0.000 |
|  | Herbivore Treatment (SL) | 1 | 879.696 | 0.000 |
|  | AA * SL | 1 | 355.240 | 0.000 |
| Transcript levels of *LOXD* | AA Treatment (AA) | 1 | 1317.841 | 0.000 |
|  | Herbivore Treatment (SL) | 1 | 6643.731 | 0.000 |
|  | AA * SL | 1 | 1342.430 | 0.000 |
| Transcript levels of *AOC* | AA Treatment (AA) | 1 | 156.479 | 0.000 |
|  | Herbivore Treatment (SL) | 1 | 3388.301 | 0.000 |
|  | AA * SL | 1 | 199.131 | 0.000 |
| JA content | AA Treatment (AA) | 1 | 5.053 | 0.044 |
|  | Herbivore Treatment (SL) | 1 | 94.803 | 0.000 |
|  | AA * SL | 1 | 3.168 | 0.100 |
| SL weight gain (%) | Genotype (G) | 1 | 239.866 | 0.000 |
|  | AA Treatment (AA) | 1 | 4.174 | 0.044 |
|  | G * AA | 1 | 1.112 | 0.294 |
| PPO activity | Genotype (G) | 1 | 74.333 | 0.000 |
|  | AA Treatment (AA) | 1 | 2.475 | 0.129 |
|  | Herbivore Treatment (SL) | 1 | 245.549 | 0.000 |
|  | G* AA | 1 | 6.024 | 0.022 |
|  | G* SL | 1 | 126.895 | 0.000 |
| Supplementary TABLE 2 Continued |  |  |  |  |
|  | AA*SL | 1 | 4.851 | 0.037 |
|  | G* AA*SL | 1 | 4.313 | 0.049 |
| POD activity | Genotype (G) | 1 | 2.298 | 0.143 |
|  | AA Treatment (AA) | 1 | 11.291 | 0.003 |
|  | Herbivore Treatment (SL) | 1 | 904.248 | 0.000 |
|  | G* AA | 1 | 5.488 | 0.028 |
|  | G* SL | 1 | 144.225 | 0.000 |
|  | AA*SL | 1 | 20.872 | 0.000 |
|  | G* AA*SL | 1 | 3.134 | 0.089 |
| PI activity | Genotype (G) | 1 | 589.392 | 0.000 |
|  | AA Treatment (AA) | 1 | 17.214 | 0.000 |
|  | Herbivore Treatment (SL) | 1 | 1592.895 | 0.000 |
|  | G* AA | 1 | 30.319 | 0.000 |
|  | G* SL | 1 | 561.824 | 0.000 |
|  | AA*SL | 1 | 17.282 | 0.000 |
|  | G* AA*SL | 1 | 30.273 | 0.000 |
